# Testosterone effect on anesthesia depth in micropenis hypospadias surgery: A prospective cohort study

**DOI:** 10.12669/pjms.42.2.13334

**Published:** 2026-02

**Authors:** Ahmet Gultekin, Ayhan Sahin, Ebru Yesildag, Ilker Yildirim

**Affiliations:** 1 Ahmet Gultekin, MD Department of Anesthesiology and Reanimation, Tekirdag Namik Kemal University Faculty of Medicine, Tekirdag, Turkiye; 2 Ayhan Sahin, MD Department of Anesthesiology and Reanimation, Tekirdag Namik Kemal University Faculty of Medicine, Tekirdag, Turkiye; 3 Ebru Yesildag, MD Department of Pediatric Surgery, Tekirdag Namik Kemal University Faculty of Medicine, Tekirdag, Turkiye; 4 Ilker Yildirim, MD Department of Anesthesiology and Reanimation, Tekirdag Namik Kemal University Faculty of Medicine, Tekirdag, Turkiye

**Keywords:** Testosterone, Micropenis, Bispectral index, Anesthesia depth, Perioperative monitoring

## Abstract

**Objective:**

To investigate prospectively the effect of testosterone treatment applied to patients with micropenis for hypospadias surgery on the depth of general anesthesia during surgery.Another objective was to make recommendations for the awareness of patients who have testosterone treatment used for sex reassignment surgery under anesthesia in the early period.

**Methodology:**

The study was a prospective cohort study conducted by the Department of Anesthesiology, Intensive Care and Pain Management of Tekirdağ Namık Kemal University Hospital between September 2024 and December 2024 (four months). We recorded the effect of intramuscular testosterone treatment administered to patients with micropenis hypospadias two weeks prior to surgery on the depth of anesthesia (1.1-1.2 MAC), as well as intraoperative changes in bispectral index (BIS), heart rate (HR), and peak airway pressure (PIP) at 5, 15, 30, 45, and 60 minutes.

**Results:**

Statistical differences were found between Group-C (Control) (n=30) and Group-T (Testosterone) (n=11). Although the depth of anesthesia was found to be between normal values for general anesthesia (BIS 40-60) in both groups, statistical differences were found between BIS, HR, and PIP values. (p< 0.05).

**Conclusion:**

For surgeries requiring testosterone treatment (such as micropenis, after gender reassignment) in the acute phase or chronic phase under general anesthesia, monitoring of anesthesia depth is recommended.

***Clinical Trial Number:*** This trial was prospectively registered on September 13, 2024 in clinicaltrials.gov with trial number “NCT06606496”.

## INTRODUCTION

Micropenis is characterized by a normally formed penis that is significantly smaller than the age- and pubertal stage-adjusted average, specifically defined as a stretched penile length (SPL) more than 2.5 standard deviations (SD) below the mean. According to global criteria, a stretched penile length less than 2cm at birth or less than 4cm beyond five years of age is typically used as a diagnostic threshold for micropenis. Normal penile development depends on fetal testicular testosterone production, its peripheral conversion to dihydrotestosterone (DHT), and subsequent interaction with androgen receptors. Etiological factors contributing to micropenis include hypothalamic-pituitary axis disorders (such as gonadotropin or growth hormone deficiencies), genetic syndromes, partial gonadal dysgenesis, testicular regression syndrome, and abnormalities in testosterone synthesis or action. The presence of concomitant findings such as hypospadias, incomplete scrotal fusion, or Cryptorchidism may be indicative of an underlying disorder of sex development (DSD).^1^

The incidence of micropenis in North America is approximately 1.5 per 10,000 male newborns. Studies have shown an average penile length of 2.6 cm in newborns for Caucasians, 2.5 cm for East Indians, and 2.3 cm for Chinese, with a slight statistical difference.^2^ Treatment may include intramuscular (IM) testosterone therapy, topical DHT (2.5% gel), and surgery for a those who do not respond to hormonal therapy and have a defective penile morphogenesis.^1^

Hypospadias is one of the most common congenital anomalies in males.^3^ Hypospadias repair often requires careful surgical techniques, and the size of the penile tissue can be a limiting factor in achieving optimal results. To address this issue, preoperative testosterone therapy increases penile length and girth, widens the glans and urethral plate, thus facilitating surgery and reducing adverse events.^4^

Monitoring the depth of anesthesia is a fundamental component of ensuring patient safety during surgical interventions. The Bispectral Index (BIS) monitor serves as a pivotal tool in this context. BIS monitoring entails the placement of electrodes on the patient’s forehead, through which the electroencephalographic (EEG) data are collected and analyzed. The resultant information, displayed numerically on the BIS monitor, offers an objective assessment of the patient’s anesthetic depth. The BIS system processes EEG signals via a proprietary algorithm to generate a numerical index ranging from 0 to 100, where 0 denotes an absence of cortical activity and 100 reflects full wakefulness. Values below 40 indicate a profound hypnotic state, while a range of 40 to 60 is considered optimal for maintaining adequate general anesthesia and preventing intraoperative awareness.^5^

Anesthetic agents induce unconsciousness by modulating hypothalamic neural circuits that are both sex-specific and hormonally regulated. Experimental studies employing various behavioral assessments have demonstrated that female rats exhibit greater resistance to volatile anesthetics compared to their male counterparts, even at equivalent anesthetic concentrations within the brain. Moreover, an anesthetic sensitivity is bidirectionally influenced by testosterone levels: conditions associated with infertility, often characterized by reduced testosterone, are linked to increased resistance to anesthesia, whereas acute exogenous administration of testosterone has been shown to enhance anesthetic sensitivity.^6^ In our study, we aimed to compare prospectively and observationally the BIS values, peak airway pressure (PIP) (cmH_2_O) and peak heart rate (/min) during general anesthesia depth monitoring in patients undergoing surgery due to hypospadias and patients receiving intramuscular (IM) testosterone due to micropenis hypospadias.

## METHODOLGY

The parents of the patients were informed about the study and their written consent was obtained. The study was a prospective cohort study and was conducted within four months.

### Sample size and group allocation:

This study did not involve random allocation. Group assignment was based on the clinical indication for preoperative testosterone therapy rather than true randomization. A total of 64 patients undergoing hypospadias surgery were screened for this study. Patients were allocated into two groups based on clinical characteristics and treatment requirements. Children diagnosed with micropenis and scheduled to receive preoperative IM testosterone were allocated to Group-T (Testosterone group), whereas children undergoing hypospadias surgery without the need for testosterone therapy were allocated to Group-C (Control group). Patients who did not meet the inclusion criteria, refused to participate, or had incomplete BIS recordings were excluded. Thus, the final analysis included 41 patients (Group-C = 30, Group-T = 11). Because the indication for testosterone treatment was determined by clinical criteria, formal randomization and allocation concealment were not applicable in this study.

### Ethical Approal:

This study was performed in line with the principles of the Declaration of Helsinki. Approval was granted by the Ethics Committee of Tekirdag Namik Kemal University Faculty of Medicine Clinical Research (Protocol Number: 2022.23.02.07) and Clinical Trials Protocol Registration and Result System registration number (NCT06606496) were obtained.

### Inclusion criteria:

- Children aged between 1-16 years scheduled for elective hypospadias surgery. ASA physical status I (healthy patients with no systemic disease). Patients in Group-T were identified with micropenis requiring preoperative intramuscular testosterone. Informed consent obtained from the patients’ guardians for participation in the study.

### Exclusion criteria:

- Patients requiring emergency surgery. Patients classified as ASA II, III, or IV (those with additional systemic diseases). Patients with BMI > 40 kg/m^2^. Patients who refused preoperative testosterone therapy. Patients or their guardians who declined participation in the study.

### Intervention:

Before each operation, leakage checks and calibrations of anesthesia devices were performed. Soda lime (Drägersorb 800 plus) (Dräger Medical, Lubeck, Germany) was used as a CO2 absorbent, Dräger Primus (Dräger Medical, Lubeck, Germany) was used as an anesthesia device, BIS™ LoC 2 channel with patient interface cable (PIC+) and BIS sensors (BIS™ pediatric sensor, Covidien Ireland Limited, Tullamore, USA) were used.

Patients scheduled for surgery due to diagnosis of micropenis hypospadias were administered a single IM dose of Sustanon (Ever Pharma Jena GmbH, Jena, Germany) two weeks before surgery, as testosterone therapy. Standard premedication (0.075 mg/kg IV (intravenous) midazolam) was administered to all patients. The sevoflurane vaporizer in the anesthesia device was adjusted to a MAC value of 1.1-1.2 according to the patient’s age. After standard general anesthesia induction (3 mg/kg propofol, 1mcg/kg fentanyl IV) and placement of a laryngeal mask (LMA) appropriate for the patient’s corrected weight, BIS monitoring was performed. BIS values were monitored within this range without intervention until the end of the case. In addition, the patient’s end tidal carbon dioxide (EtCO2) was adjusted to 30-35 mmHg throughout the case and BIS, HR and PIP values were recorded at 5, 15, 30, 45 and 60 minutes.

**Fig.1:**
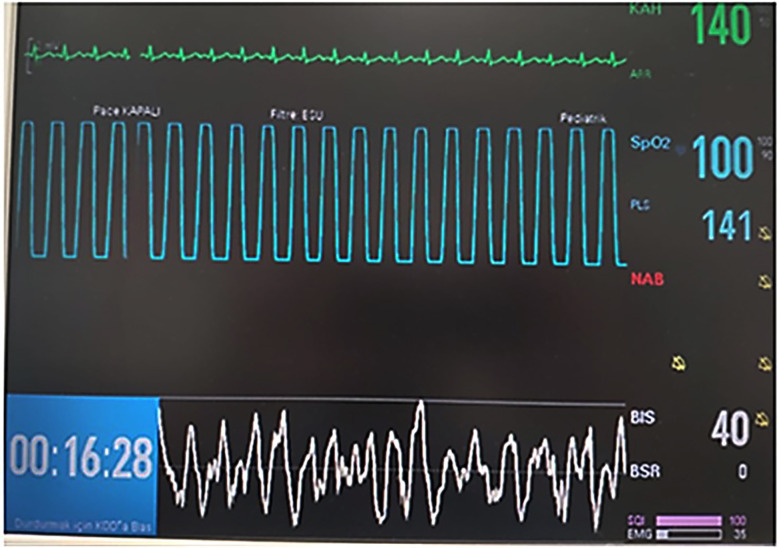
BIS monitoring.

Patients were monitored with electrocardiogram (ECG), heart rate (HR), and peripheral oxygen saturation (SpO2) before the operation. In order to adjust the age-appropriate MAC (minimum alveolar concentration) and tidal volume in the anesthesia device before induction, the patient’s age and ideal weight were entered and follow-up alarm limits were set. All patients were preoxygenated with 4 L/minutes and 100% oxygen for three minutes before induction. Following the induction of anesthesia, LMA Proseal was applied according to the patient’s weight and BIS monitoring was added to the patient according to the instructions (Fig.1).

### Consent for Publication:

All participants provided written informed consent for study participation and publication, approved by the institutional ethics committee.

### Statistical Analysis:

**Fig.2:**
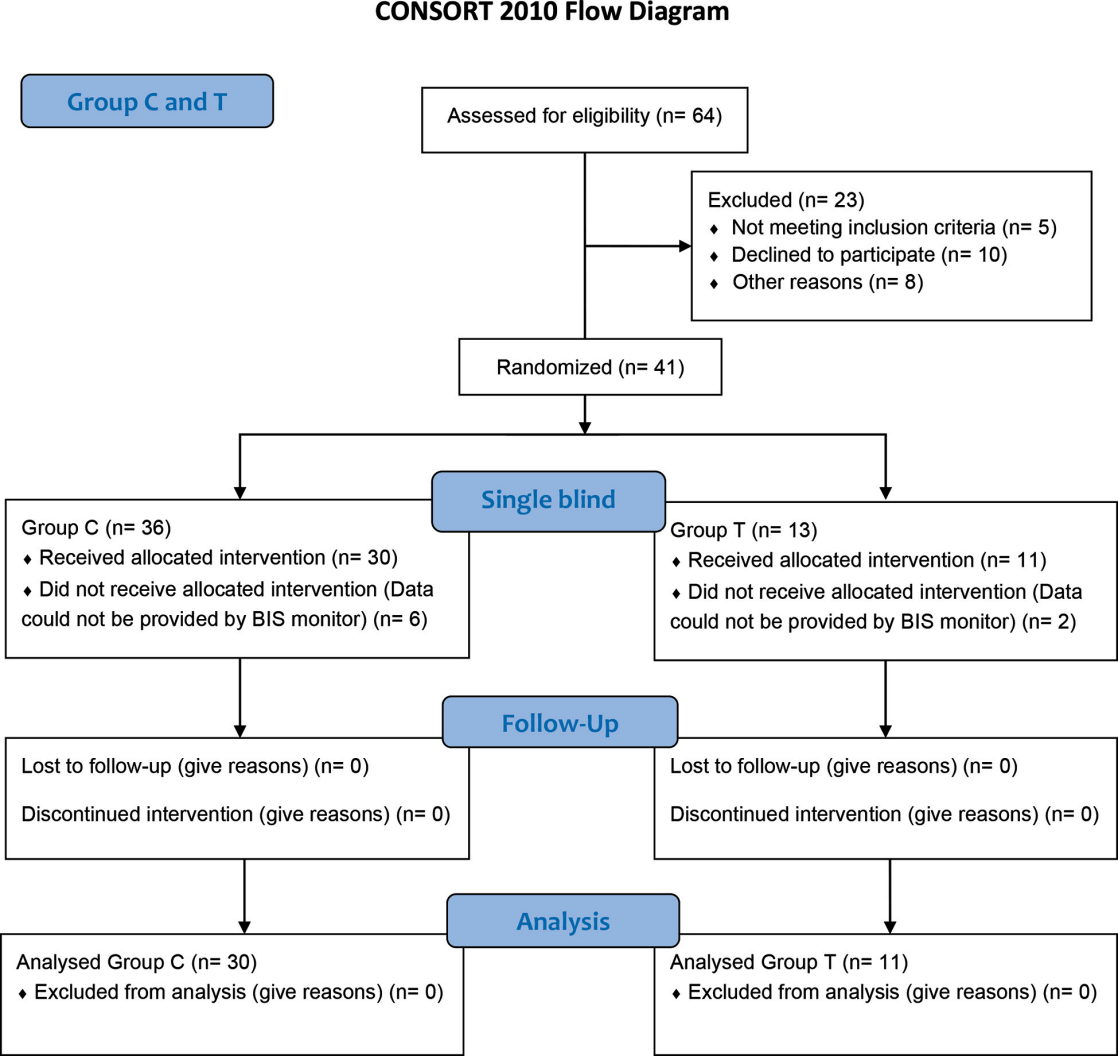
Flow diagram of the patients who participated in the study.

A total of 64 patients were screened, of whom 23 were excluded due to not meeting the inclusion criteria, refusal to participate, or incomplete BIS recordings. Thus, the final analysis included 41 patients (Group-C = 30, Group-T = 11) (Fig.2).

All statistical analyses were performed using SPSS Statistics for Windows, Version 21.0 (IBM Corp., Armonk, NY, USA). Normality of continuous variables was assessed by the Shapiro-Wilk test. Continuous data were expressed as mean ± standard deviation (SD) for normally distributed variables and as median (interquartile range, IQR) for non-normally distributed variables. Categorical variables were expressed as numbers and percentages. To ensure comparability between groups, MAC and EtCO_2_ values were analyzed and found to be statistically similar (p > 0.05). Age and body weight significantly differed between groups (p < 0.05), these variables were considered as potential covariates in the analysis.

Between-Group-Comparisons of continuous variables were conducted using Welch’s t-test or Mann-Whitney U test, depending on the distribution and equality of variances. For repeated measurements of BIS, HR, and PIP values at 5, 15, 30, 45, and 60 minutes, repeated measures ANOVA was used for normally distributed data with Greenhouse-Geisser correction when sphericity was violated, and the Friedman test was used for non-normally distributed data. Where appropriate, ANOVA was additionally performed to adjust for age and weight as covariates. Post-hoc pairwise comparisons were adjusted using the Bonferroni correction.

Surgical duration between groups was compared using the Mann-Whitney U test is used due to distribution’s skew. Statistical significance was set at p < 0.05. All analyses were reviewed and confirmed by an independent biostatistician, and a certificate of statistical analysis was provided.

## RESULTS

A total of 64 patients underwent hypospadias surgery. Of our patients, 23 did not meet the study criteria, declined participation, or were excluded due to insufficient BIS monitoring data, resulting in a final sample of 41 patients (Group-C = 30, Group-T = 11). Patients who had received preoperative testosterone therapies were identified only after surgical and anesthesia data collection was completed. Therefore, group allocation was based on clinical characteristics rather than randomization, and the study followed a non-randomized prospective cohort design (Fig.2).

In order to ensure standardization of the groups, statistically similar groups were provided in terms of MAC and EtCO_2_ values (p> 0.05), and in terms of age, since the difficulty in micropenis surgeries occurs at among younger ages, there was a difference in terms of age and weight (p < 0.05). Statistically significant differences were found between our groups in terms of BIS values (although they remained between the values that would be considered normal (40-60) for general anesthesia), PIP, heart rate, and surgery duration. (p < 0.05) (Table-I).

**Table-I:** Demographic data and findings.

Variable	Time (minute)	Group-C (n=30) mean ± standard deviation (SD) and IQR	Group-T (n=11) mean ± standard deviation (SD) and IQR	P value
Age (years)	—	5.4 ± 3.0	2.3 ± 1.6	<0.001*
Weight (kg)	—	22.9 ± 10.3	14.4 ± 3.7	<0.001*
BIS	5	38.6 ± 9.3	52.2 ± 7.5	<0.001#
	15	39.4 ± 9.4	51.9 ± 9.9	0.001#
	30	38.2 ± 8.3	50.0 ± 10.1	0.001#
	45	38.2 ± 8.5	49.6 ± 7.7	<0.001#
	60	40.1 ± 12.1	52.1 ± 7.6	0.011#
Heart Rate (/min)	5	118.5 ± 26.7	138.8 ± 15.1	0.023#
	15	125.7 ± 27.6	148.6 ± 13.7	0.013#
	30	114.6 ± 25.6	135.1 ± 16.4	0.019#
	45	108.3 ± 24.4	128.2 ± 9.6	0.013#
	60	105.3 ± 29.5	124.9 ± 6.1	0.049#
PIP (cmH_2_O)	5	12.5 ± 2.2	15.8 ± 2.8	<0.001#
	15	12.3 ± 2.1	16.4 ± 2.6	<0.001#
	30	13.0 ± 2.4	15.6 ± 2.3	0.005#
	45	13.1 ± 2.6	16.3 ± 2.3	0.001#
	60	13.6 ± 2.9	16.3 ± 2.2	0.017#
MAC (min-max)	5	1.1 (1.1-1.2)	1.1 (1.1-1.2)	0.499†
	15	1.2 (1.1-1.2)	1.2 (1.1-1.2)	0.966†
	30	1.1 (1.1-1.2)	1.1 (1.1-1.2)	0.145†
	45	1.2 (1.1-1.2)	1.2 (1.1-1.2)	0.070†
	60	1.1 (1.1-1.2)	1.2 (1.1-1.2)	0.686†
EtCO_2_ (mmHg)	5	32.4 ± 4.8	33.0 ± 1.7	0.706†
	15	31.5 ± 4.6	31.8 ± 1.3	0.843†
	30	31.1 ± 4.6	32.7 ± 0.9	0.241†
	45	31.1 ± 4.7	32.9 ± 1.7	0.215†
	60	30.4 ± 6.1	33.0 ± 1.3	0.195†
Surgery time (minute)	—	61.9 ± 18.1	72.6 ± 7.0	0.01†

* Welch’s t-test, #Repeated measures ANOVA (for normally) / Friedman for repeated measures (for non-normally, †Mann-Whitney U test, MAC = minimum alveolar concentration; BIS = bispectral index; PIP = peak inspiratory pressure; EtCO_2_ = end-tidal CO_2_, min: minumum, max: maksimum.

## DISCUSSION

In this prospective cohort study, we found that children who received preoperative intramuscular testosterone had significantly higher BIS values, heart rates, and peak inspiratory pressures during sevoflurane anesthesia, despite similar MAC and EtCO_2_ values between the groups. These findings suggest that testosterone administration may reduce anesthetic depth or modify autonomic responses during general anesthesia. Our results indicate a measurable perioperative physiological effect associated with testosterone exposure in prepubertal patients.

The anesthesiologist should be knowledgeable about the abuse of anabolic steroids, their possible side effects, and the perioperative risks associated with the use of these drugs.^7^ The actions of neurosteroids on GABA-A receptors are sexually dimorphic. It is therefore possible that the direct actions of sex hormones on the GABA-A receptor contribute to their effects on anesthetic sensitivity.^8^ The effects of exogenous testosterone administration on brain wave profiles were examined in both castrated and non-castrated rats. Testosterone administration resulted in increases in low-frequency (delta and theta) and high-frequency (beta and gamma) activity.

These changes suggest that testosterone has an effect on neurological functions.^9^ Niu et al. studied the neurotoxic effects of testosterone in developing brains and indicated that testosterone may have an effect on the anesthesia-sensitive brain.^10^ Given that nearly all anesthetic agents possess GABA agonist or NMDA antagonist properties, similar to ethanol, it is plausible that neonatal anesthesia exposure may influence perinatal hormonal fluctuations, and this interaction may manifest behaviorally in similar ways, such as the emergence of opposite-sex sex-typical behaviors (female aggression), the decline of sex-typical behaviors (female sexual receptivity), or the exacerbation of sex-typical behaviors (male aggression).^11^ There are studies showing that estrogen^12^, progesterone,^13^,^14^ estradiol and testosterone^15^ levels affect the need for anesthetic agents. Sex hormones, particularly testosterone and estrogen, play crucial roles in modulating neuronal excitability and anesthetic sensitivity. The anesthesiologist should therefore be aware not only of their physiological roles, but also of potential alterations induced by exogenous administration. Our study was conducted to determine the effect of testosterone treatment on the depth of peroperative anesthesia and vital parameters, and to contribute to the evaluation of the adequacy of anesthetic agents.

Do et al.^16^ showed in their meta-analysis that preoperative androgen stimulation (with testosterone and others) for hypospadias surgery was not associated with postoperative surgical complications as a result of promoting penile growth. Furthermore, findings from the study by Yang et al.^17^ and the review by Jevtovic-Todorovic 18 titled *“Testosterone: Much More for the Brain Than a Sex Hormone”* indicates that exogenous testosterone administration to immature mouse pups during sevoflurane exposure led to elevated brain testosterone levels and exerted neuroprotective effects. Specifically, testosterone attenuated tau protein phosphorylation, inhibited the activation of glycogen synthase kinase-3β (GSK-3β) and its interaction with tau, counteracted the sevoflurane-induced suppression of neuronal activity, and mitigated associated cognitive deficits. These results suggest that testosterone is critically involved in modulating multiple signaling pathways essential for neuroprotection and the maintenance of normal neuronal circuit function in the developing male brain. It also suggests that low testosterone levels in very young mammals may contribute, at least in part, to the sensitivity of the neonatal male brain to anesthesia.

Analysis of multiple behavioral and neurocognitive measures in healthy human volunteers who underwent a precisely controlled anesthetic exposure in the absence of surgical stimuli confirmed that human females are also more resistant to volatile anesthetics.^19^ The female brain is more resistant to the hypnotic effects of anesthetics, and anesthetic resistance is suggested to be of hypothalamic origin. Awareness is found to be higher under anesthesia in women and recovery is faster than in men.^20^ However, this increased resistance is not readily detectable on the EEG. The effects of postpubertal brain circuit maturation and/or sex hormone levels on stable anesthesia sensitivity are largely unknown. Studies in mice have shown that acute anesthesia sensitivity of testosterone is mediated by its conversion to estradiol by the aromatase enzyme.

The results suggest that there are clear behavioral differences between the sexes in mice and humans, but that conventional EEG measurements do not reveal sex differences in anesthetic depth in either species. Unlike this study, our patients were pre-pubertal and also in the BIS monitoring, BIS values were significantly higher in the testosterone group (below 1.1-1.2 MAC). Increased BIS values and higher hemodynamic parameters in the testosterone-treated group in our study may reflect elevated sympathetic activity or reduced anesthetic depth due to hormonal modulation of GABAergic transmission. In addition, the heart rate and PIP values of the patients we followed were also significantly higher in the testosterone treatment group.

The difference in the operation time is due to the testosterone group having penile dimensions considered micropenis and being younger. However, in a study conducted by Duymaz et al.^15^ which is parallel to our study, it was stated that daily changing testosterone levels are parallel to the drug levels required for sedation, and it was added that larger-scale and planned studies are required. Taken together, these findings highlight that preoperative or therapeutic testosterone exposure can significantly influence anesthetic depth and physiological responses, warranting careful intraoperative monitoring in pediatric and hormonal therapy populations.

### Strength of the Study:

To our knowledge, this is the only clinical study evaluating intraoperative depth of anesthesia using BIS in pre-pubescent patients receiving testosterone therapy. This study contributes to the literature by demonstrating that even a single preoperative testosterone dose may influence anesthetic sensitivity and autonomic responses in children. Clinically, these findings highlight the importance of careful intraoperative monitoring and individualized anesthetic management in patients receiving hormonal therapy. The strength of our study lies in its prospective design and standardized anesthetic protocol. Future studies with larger samples, multiple hormone regimens, and complementary depth-of-anesthesia monitoring modalities are warranted to confirm these preliminary findings.

### Limitations:

Increasing the sample size and diversity of groups could have yielded more accurate results. Another situation we experienced in the study was the difficulties in obtaining data in BIS monitoring, and we recommend that methods other than BIS be considered in terms of depth of anesthesia. It is also necessary to add to our limitations that all patients in the testosterone group were administered a single dose of testosterone (IM) equally.

## CONCLUSION

Our study yielded opposite results compared to the studies conducted on testosterone and anesthesia sensitivity in the literature. Although we think that the effect here is mainly due to exogenous testosterone, we also think that it may be due to the physiological state of the micropenis. The presence of pre-pubertal patients in our study prevented it from being affected by hormonal fluctuations, and as a result, it should be considered that our findings may reflect the real results. In addition to the treatment of micropenis hypospadias, it is also important to monitor the depth of anesthesia for surgeries that individuals who have to use regular testosterone treatment due to gender reassignment, especially in the first period of their lives and in the future.
